# A case-control study on the effects of incomplete, one, and more than one dexamethasone course on acute respiratory problems in preterm neonates born between 28^0^ and 36^6^ weeks of gestation

**DOI:** 10.1186/s12884-022-05209-6

**Published:** 2022-11-28

**Authors:** Saifon Chawanpaiboon, Julaporn Pooliam, Monsak Chuchotiros

**Affiliations:** 1 Division of Maternal-Fetal Medicine, Department of Obstetrics & Gynaecology, Faculty of Medicine, Siriraj Hospital, Mahidol University, Bangkok, 10700 Thailand; 2 Clinical Epidemiological Unit, Office for Research and Development, Faculty of Medicine, Siriraj Hospital, Mahidol University, Bangkok, 10700 Thailand

**Keywords:** Dexamethasone, Incomplete course, Very preterm, Moderate preterm, Late preterm, Respiratory problems

## Abstract

**Objective:**

To compare the effects of an incomplete course and more than 1 course of dexamethasone, relative to a control of a single complete course, on foetal respiratory problems and other adverse outcomes of preterm birth.

**Methods:**

This was a retrospective chart review of 1800 women with preterm delivery. Data were collected on newborns whose mothers administered 1 full course of dexamethasone (916/1800; 50.9%), a partial course (716/1800; 39.8%) and more than 1 course (168/1800; 9.3%). Demographic data and adverse maternal and neonatal outcomes were recorded.

**Results:**

Preterm singleton newborns whose mothers received several steroid hormone courses were significantly more likely to have adverse outcomes than newborns of mothers given 1 course. The negative outcomes were the need for positive pressure ventilation ([aOR] 1.831; 95% CI, (1.185,2.829); *P* = 0.019), ventilator support ([aOR] 1.843; 95% CI, (1.187,2.861); *P* = 0.011), and phototherapy ([aOR] 1.997; 95% CI, (1.378,2.895); *P* <  0.001), transient tachypnoea of the newborn ([aOR] 1.801; 95% CI, (1.261,2.571); *P* = 0.002), intraventricular haemorrhage ([aOR] 2.215; 95% CI, (1.159, 4.233); *P* = 0.027), sepsis ([aOR] 1.737; 95% CI, (1.086, 2.777); *P* = 0.007), and admission to neonatal intensive care ([aOR] 1.822; 95% CI, (1.275,2.604); *P* = 0.001). In the group of very preterm infants, newborns of mothers administered an incomplete course had developed respiratory distress syndrome (RDS) ([aOR] 3.177; 95% CI, (1.485, 6.795); *P* = 0.006) and used ventilatory support ([aOR] 3.565; 95% CI, (1.912, 6.650); *P* <  0.001) more than those of mothers receiving a single course.

**Conclusions:**

Preterm singleton newborns whose mothers were given multiple courses of dexamethasone had an increased incidence of RDS and other adverse outcomes than those of mothers receiving a full course. However, very preterm newborns whose mothers were administered 1 full dexamethasone course had a significantly lower incidence of RDS than those whose mothers were given partial courses.

## Introduction

Global estimated of live preterm birth was 14·84 million (12·65 million-16·73 million) in 2014 [1]. Each year, of 4 million infant deaths, over 1 million are preterm [2]. The major causes of death are respiratory distress syndrome (RDS), an underdeveloped lung problem or insufficient surfactant production in preterm infants [3]. Babies with advanced gestational age have better lung development, reducing the RDS incidence.

Liggins first investigated the steroid hormones known as glucocorticoids to determine their ability to prevent RDS [4]. Glucocorticoids have been widely used to prevent RDS in the infants of mothers at risk of preterm labour and have effectively reduced infant morbidity and mortality [5].

Glucocorticoids have been reported to benefit preterm infants’ brains and bowels because the steroids help prevent intraventricular haemorrhage (IVH) and necrotising enterocolitis (NEC) [6]. In preterm singleton babies, glucocorticoids reduce the risk of RDS by 38%, IVH by 48%, NEC by 50% and stillbirth by 25% [7]. Institutions worldwide (such as the US National Institutes of Health, the Royal College of Medicine, the American College of Obstetricians and Gynaecologists [3] and others [8–10]) recommend glucocorticoid administration in pregnant women at risk of preterm delivery to prevent RDS in their newborns.

Two types of steroid hormones are effective for lung development:

- Betamethasone (betamethasone-acetate/betamethasone-phosphate) (12 mg intramuscularly; 2 times; 24 hours apart), and
- Dexamethasone (dexamethasone-phosphate (6 mg intramuscularly; 4 times; 12 hours apart).

The dosage is based on the concentration of glucocorticoids in the infant compared with the physiological stress doses of cortisol detected in preterm infants with RDS [11]. Administration of both glucocorticoids at the recommended doses resulted in binding to 75 to 80% of alveolar glucocorticoid receptors, sufficient to stimulate lung function [12]. Increased glucocorticoid dosage or frequency does not affect lung function but can lead to potentially harmful effects. They are adrenal insufficiency, foetal growth restriction and the risk of infection due to immunosuppression [13].

Short-term use of steroids positively affects the development of the lungs, preventing brain and gut complications. However, it may affect the development of the tissues and organs of the foetal body (foetal programming) in the long term [14–16]. Some studies found that premature newborns have alterations in the hypothalamic-pituitary-adrenal axis [17, 18], abnormal foetal metabolism [19, 20], high blood pressure [19, 21], and delayed myelination in the central nervous system, resulting in abnormal psychomotor development [22–24].

Although it has been almost 50 years since glucocorticoids were first used, there is a lack of long-term follow-up of children. Animal studies have found that betamethasone had a greater effect on brain development in rats [25, 26] and abnormalities of the insulin resistance system over the age of 30, which in turn increased the risk of diabetes [27]. An increasing number of animal and human studies have found that multiple courses of steroids may slow the development of infants [28–31] and cerebral palsy in children 2 years of age delivered from mothers receiving 1 or more courses of betamethasone [32]. However, the condition was not found in children delivered from mothers administered a half course of betamethasone [33], which was also as effective in stimulating lung development as a full course [34, 35].

Thailand has long used steroid hormones for RDS prevention in premature babies. Some pregnant women come to the hospital in emergency conditions and are delivered soon after admission, so less than a complete course of steroids can be administered. Some expecting mothers have a hospital stay longer than 1 week, which may require another rescue course of dexamethasone to improve lung function. We are interested in conducting a comparative study of dexamethasone in doses less than 1 full course versus more than 1 course, relative to a control group of a single complete course, to determine the effects on foetal respiratory problems and other adverse outcomes of preterm birth and mothers.

## Materials and methods

This retrospective chart- reviewed study was conducted in the statistical unit of the Department of Obstetrics and Gynecology of the Faculty of Medicine, Siriraj Hospital. Before starting this research, its protocol was approved by the Ethics Committee of the Faculty of Medicine Siriraj Hospital (Si297/2021). The work was also registered in the Thai Clinical Trials Registry (TCTR 20210425002). The authors thank the Faculty of Medicine Siriraj Hospital, Mahidol University, for its financial support ([IO]R016433028).

Our prior pilot study showed that 68% of preterm infants with RDS had taken 1 full course of dexamethasone. In another analysis of preterm and full-term newborns, 50% of preterm infants with RDS had taken either an incomplete course or more than 1 course. The sample size calculation was based on a statistical significance of 0.01 (type I error = 1%; 2-sided) and power of the test = 95% (type II error = 5%). The size of each group of neonates with RDS (263 infants) was calculated using the nQuery Advisor program.

Our pilot study determined that 15% of preterm infants suffered from RDS. Therefore, 263 × 100/15 = 1753 preterm infants were needed for the study. This figure was rounded to 1800 preterm births.

Data related to 1800 pregnant women with preterm delivery between 2016 and 2020 were collected from hospital records. Baseline characteristics of the women were collected. Laboratory blood test results, number of antenatal visits, delivery route, gestational age at delivery, neonatal and maternal complications, neonatal weight, and APGAR scores were recorded.

The primary outcome of this study was the effects of different doses/courses of dexamethasone on RDS in preterm infants during 28^0^–36^6^ weeks of gestation. The secondary outcomes were the effects on RDS of a single course of dexamethasone (4 times; 12 hours apart), a partial course, and more than 1 course. Analyses were performed on 5 subgroups:

- preterm singletons during 28^0^–36^6^ weeks of gestation
- preterm twins during 28^0^–36^6^ weeks of gestation
- very preterm infants (28^0^–31^6^ weeks of gestation)
- moderate preterm infants (32^0^–33^6 ^weeks of gestation)
- late preterm infants (34^0^–36^6^ weeks of gestation).

Details relating to the following were collected for each subgroup: APGAR scores < 7, positive pressure ventilator (PPV) of newborns, neonatal intensive care unit (NICU) admission, respiratory support of newborns, RDS, transient tachypnoea of the newborn (TTNB), apnoea, IVH, NEC, early onset neonatal sepsis and pneumonia. Maternal complications during the postpartum period and the length of hospital stay were also analysed. The final diagnosis of acute respiratory problem was made before the newborn was discharged from the hospital.

### Definition of acute respiratory problems

- “RDS” is defined as infants breathing more than 60 times per minute or having nasal flaring, expiratory grunting, or chest wall retractions, and receiving oxygenation for over 2 hours postpartum and hypoxemia which defined by a PaO_2_/FiO_2_ ratio ≤ 200 mmHg, and no evidence of left atrial hypertension or a pulmonary capillary pressure <  18 mmHg (if measured) to rule out cardiogenic edema [36, 37].
- “Bronchopulmonary dysplasia (BPD) is defined as very preterm infants with chest x-ray presented of persistent lung parenchyma disease and preterm infants born after 35^6^ weeks of gestation requiring oxygen or respiratory support to maintain SpO2 in the 90–95% range for at least 3 days. BPD does not include infants requiring ventilator due to central hypoventilation or airway disease [38].
- Apnoea of prematurity is defined as respiratory pauses > 20 seconds or pauses < 20 seconds that are associated with bradycardia (< 100 beats/minute), central cyanosis, and/or oxygen saturation < 85% in neonates born at < 37 weeks gestation and with no underlying disorders causing apnoea [39].
- Transient tachypnoea of the newborn is defined as infants with tachypnoea, retractions, grunting, and nasal flaring. Diagnosis is suspected when there is respiratory distress shortly after birth and is confirmed by chest x-ray. Recovery usually occurs within 24 hours [40].
- Persistent tavhypnoea of the newborn is defined as infants with crackles, retractions, digital clubbing, failure to thrive, or respiratory failure; hypoxemia and diffuse radiological abnormalities present at diagnosis with a minimum duration of 4 weeks [41].

### Statistical analysis

Statistical analyses were performed using PASW Statistics for Windows (version 18.0; SPSS Inc., Chicago, IL, USA). Demographic data were analysed using descriptive statistics. Categorical data are presented as numbers and percentages, and continuous data are presented as the means ± standard deviations or as medians and ranges. Baseline data (qualitative parameters and adverse maternal and newborn outcomes) were compared using the chi-squared test with adjusted for multiple comparison using the Bonferroni correction.

For non-normally distributed quantitative variables, the Kruskal-Wallis test was used to analyse the difference between groups and Dunn’s test was used for pairwise comparison. Multivariable logistic regression models were used to calculate the adjusted odds ratios and 95% confidence intervals (95% CI) for the association between steroid groups and adverse outcomes after adjusting for covariate (maternal age, BMI, morbidities). *P*-values less than 0.05 was considered statistically significant.

## Results

Of the 1800 pregnant women at risk of preterm delivery and receiving steroid administrations between 2016 and 2020, 50% (900/1800) were labourers, and 26.6% (479/1800) were housewives. Most pregnancies were singletons (88.0%; 1550/1800) and in the first pregnancy (42.9%; 773/1800). The mean gestational age at delivery was 34.4 ± 2.1 weeks of gestation. A single steroid hormone course was administered to 50.9% (916/1800) of the cases, an incomplete course to 39.8% (716/1800), and more than 1 course to 9.3% (168/1800). Most women (47.4%; 854/1800) delivered within 48 hours after steroid administration (Table 1). The mean body weight of the infants was 2178.6 ± 557.4 g. Approximately one-fifth (16.9%; 341/1800) had an APGAR score at 1 minute < 7 (Table 1).

**Table 1:** Maternal demographic data

**Mother**	**Total** **(*N*** **= 1800)**	**< 1 course** **(*N*** **= 716**	**1 course** **(*N*** **= 916)**	**> 1 course** **(*N*** **= 168)**
Occupation				
Housewife	479 (26.6%)	174 (24.4%)	264 (28.9%)	41 (24.4%)
Labourer	900 (50.0%)	393 (54.9%)	427 (46.6%)	80 (47.6%)
Merchant	112 (6.2%)	46 (6.5%)	61 (6.6%)	5 (3.0%)
Government officer	138 (7.7%)	51 (7.1%)	69 (7.6%)	18 (10.7%)
Self-employed	77 (4.3%)	22 (3.0%	41 (4.5%)	14 (8.3%)
Student	28 (1.6%)	14 (1.9%)	11 (1.2%)	3 (1.8%)
Other	58 (3.2%)	11 (1.5%)	41 (4.4%)	6 (3.6%)
Unknown	8 (0.4%)	5 (0.7%)	2 (0.2%)	1 (0.6%)
Monthly income (Baht)				
< 5000	31 (1.7%)	17 (2.3%)	12 (1.3%)	2 (1.2%)
5000–9999	232 (12.9%)	108 (15.1%)	113 (12.3%)	11 (6.5%)
10,000–19,999	497 (27.6%)	226 (31.6%)	227 (24.8%)	44 (26.2%)
20,000–49,999	440 (24.4%)	157 (21.9%)	235 (25.6%)	48 (28.6%)
≥ 50,000	127 (7.1%)	35 (4.9%)	69 (7.6%)	23 (13.7%)
Unknown	473 (26.3%)	173 (24.2%)	260 (28.4%)	40 (23.8%)
Pregnancy				
Singleton	1584 (88.0%)	673 (94.0%)	783 (85.5%)	128 (76.2%)
Twins	216 (12.0%)	43 (6.0%)	133 (14.5%)	40 (23.8%)
Age (years)	30.3 ± 6.7	30.0 + 6.6	30.5 + 6.6	30.9 + 6.6
Body mass index (kg/m^2^)	27.1 ± 5.0	27.3 + 4.8	27.1 + 4.9	26.4 + 5.4
Gravida				
1	773 (42.9%)	286 (39.9%)	407 (44.4%)	80 (47.6%)
≥ 2	1027 (57.1%)	430 (60.1%)	509 (55.6%)	88 (52.4%)
Parity				
0	971 (53.9%)	352 (49.2%)	520 (56.8%)	99 (58.9%)
≥ 1	829 (46.1%)	364 (50.8%)	396 (43.2%)	69 (41.1%)
Abortion				
0	1409 (78.3%)	565 (78.9%)	712 (77.7%)	132 (78.6%)
≥ 1	391 (21.7%)	151 (21.1%)	204 (22.3%)	36 (21.4%)
Gestational age at delivery (weeks of gestation)	34.4 ± 2.1	34.1 + 1.5	34.9 + 1.4	33.6 + 1.5
Number of pregnant women with re-admission	475 (26.4%)	31 (4.3%)	354 (42.0%)	90 (53.6%)
Gestational age at readmission (weeks of gestation)	31.5 ± 3.9	32.2 + 2.6	32.5 + 2.0	30.2 + 1.9
Underlying disease				
Diabetes mellitus (DM)	29 (1.6%)	11 (1.5%)	15 (1.6%)	3 (1.8%)
Hypertension	107 (5.9%)	37 (5.2%)	50 (5.5%)	20 (11.9%)
Others	173 (9.6%)	64 (8.9%)	90 (9.8%)	19 (11.3%)
Number of antenatal visits				
0	61 (3.4%)	42 (5.8%)	16 (1.7%)	3 (1.8%)
< 5	250 (13.9%)	108 (15.1%)	114 (12.5%)	28 (16.7%)
5–10	1368 (76.0%)	513 (71.5%)	723 (78.9%)	132 (78.6%)
> 10	121 (6.7%)	53 (7.4%)	63 (6.9%)	5 (2.9%)
Time from starting steroids to delivery				
≤ 48 hours	854 (47.4%)	698 (97.5%)	151 (16.5%)	5 (3.0%)
> 48 hours–7 days	385 (21.4%)	9 (1.3%)	369 (40.3%)	7 (4.2%)
> 7 days–14 days	178 (9.9%)	5 (0.7%)	142 (15.5%)	31 ((18.5%)
> 14 days–21 days	122 (6.8%)	1 (0.1%)	88 (9.6%)	33 (19.5%)
> 21 days	261 (14.5%)	3 (0.4%)	166 (18.1%)	92 (54.8%)
Delivery				
Spontaneous vaginal delivery	725 (40.3%)	322 (45.0%)	360 (39.3%)	43 (25.6%)
Caesarean section	1063 (59.1%)	390 (54.5%)	549 (59.9%)	124 (73.8%)
Vacuum extraction	10 (0.6%)	3 (0.4%)	7 (0.8%)	0 (0)
Forceps extraction	2 (0.1%)	1 (0.1%)	0 (0)	1 (0.6%)
Puerperal infection	13 (0.7%)	5 (0.7%)	6 (0.7%)	2 (1.2%)
Length of hospital stay (days)	6 (2–143)	5 (2–31)	9 (2–57)	20 (2–143)
**Newborn**	**All** **(*N*** **= 2015)**	**< 1 course** ***N*** **= 759**	**1 course** ***N*** **= 1049**	**> 1 course** ***N*** **= 207**
Mean neonatal weight (grams)	2178.6 ± 557.4	2283.3 + 570.1	2153.6 + 543.0	1921.6 + 481.2
Apgar at 1 min < 7	341 (16.9%)	114 (15%)	177 (16.9%)	50 (24.2%)
Apgar at 5 min < 7	94 (4.7%)	39 (5.1%)	42 (4%)	13 (6.3%)
Haemoglobin (Hb) (g/dL)	16.9 ± 3.2	16.9 + 3.3	16.8 + 3.1	16.8 + 3.7
Haematocrit (Hct) %	48.3 ± 8.0	48.6 + 8.0	48.3 + 7.9	47.3 + 7.7
Microbilirubin (MB) (mg/dL)	8.5 ± 3.4	8.9 + 3.6	8.4 + 3.3	7.4 + 2.5
Sex				
Male	1037 (51.5%)	401 (52.8%)	540 (51.5%)	96 (46.4%)
Female	978 (48.5%)	358 (47.2%)	509 (48.5%)	111 (53.6%)

Data was presented as mean ± standard deviation (SD), median (minimum, maximum), and number (%)

**Fig. 1:**
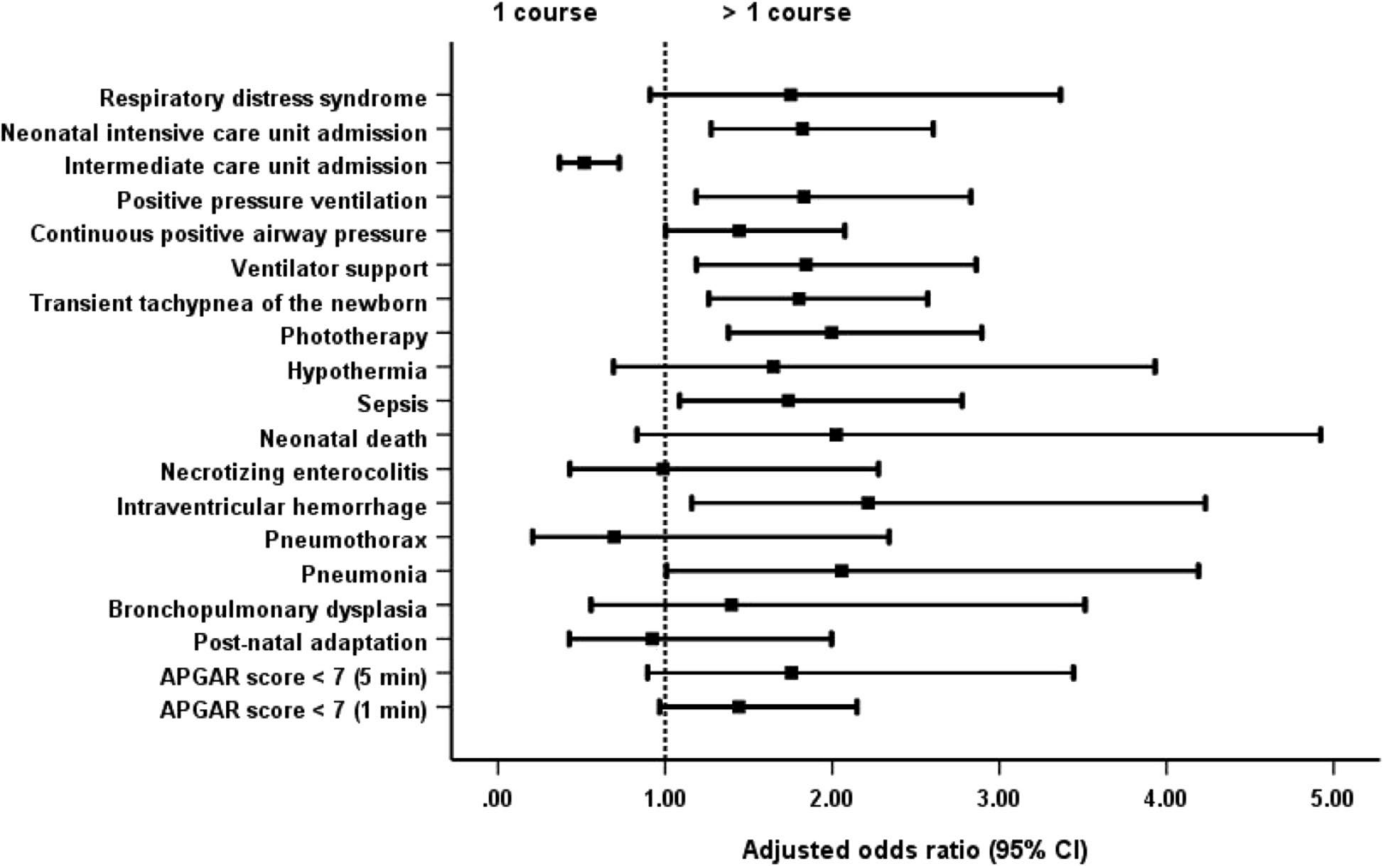
Comparison of neonatal and maternal outcomes with the administration of 1 and >  1 course of dexamethasone. *For overall analysis, one course of dexamethasone has better neonatal and maternal outcomes than more than 1 course of dexamethasone administration

**Fig. 2:**
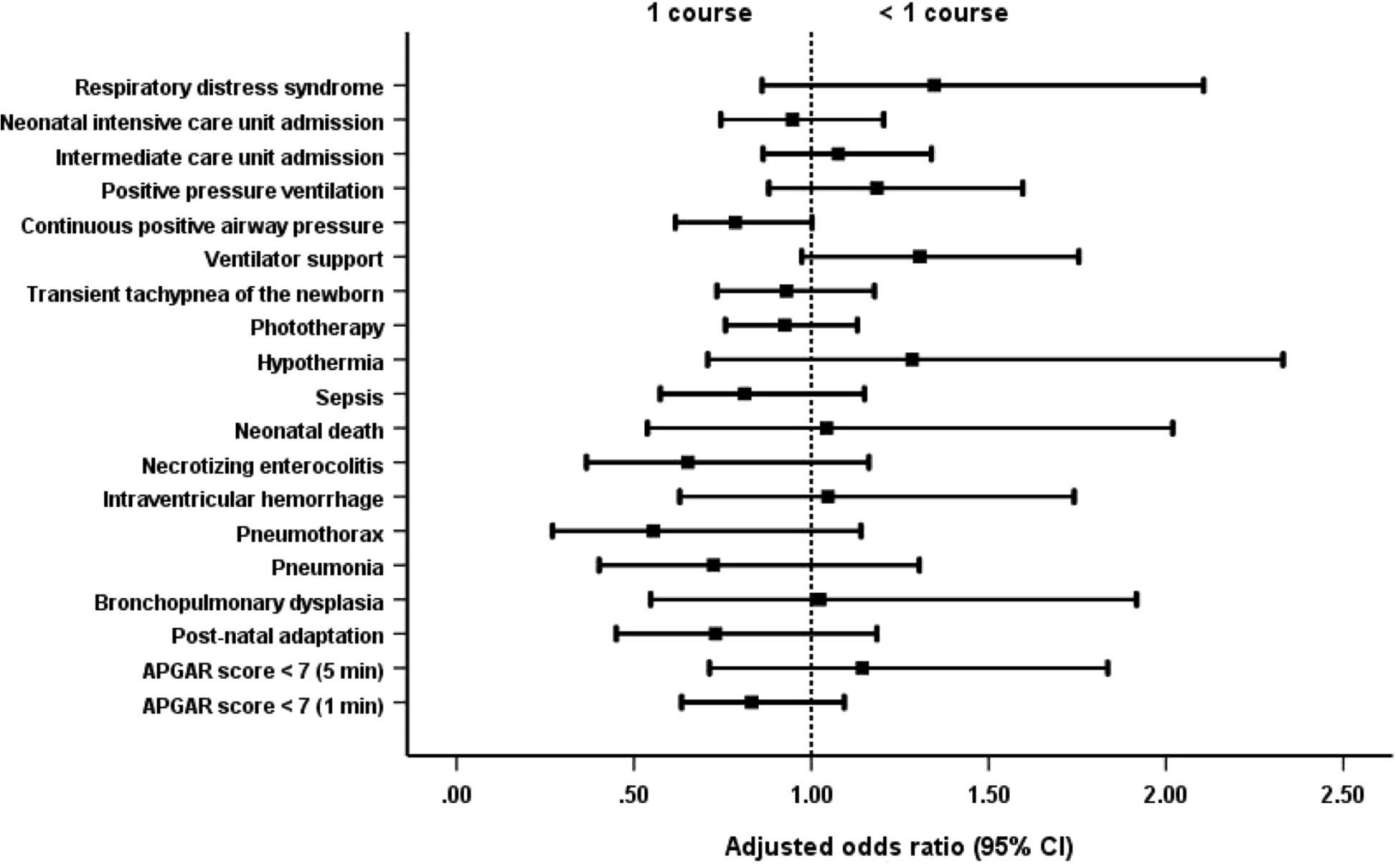
Comparison of neonatal and maternal outcomes with the administration of 1 and < 1 course of dexamethasone. * For overall analysis, neonatal and maternal outcomes are not different between full and partial course of dexamethasone administration

Newborns of mothers given more than 1 course of steroids were significantly more likely to have adverse outcomes than newborns of mothers receiving 1 complete course. The outcomes were the development of TTNB, IVH and sepsis; admission to the NICU; the need for PPV, ventilator support and phototherapy; and a prolonged hospital stay (Table 2; Fig. 1). Newborns of mothers who received less than a full course of steroids had a significantly shorter length of hospital stay than neonates whose mothers were administered a complete course without difference of others adverse neonatal outcomes (Table 2; Fig. 2).

**Table 2:** Neonatal and maternal outcomes

Outcomes	Total (***N*** = 1800)	< 1 course (***n*** = 716): (1)	1 course (***n*** = 916): (2)	> 1 course (***n*** = 168): (3)	***P*** value	Adjusted odds ratio (95% CI) for < 1 course	Adjusted odds ratio (95% CI) for > 1 course
**Neonatal**							
APGAR score < 7 (1 min)	301 (16.7%)	104 (14.5%)	157 (17.1%)	40 (23.8%)	0.013^ab^	0.832 (0.634–1.093)	1.441 (0.967–2.148)
APGAR score < 7 (5 min)	85 (4.7%)	34 (4.7%)	39 (4.3%)	12 (7.1%)	0.269	1.144 (0.713–1.836)	1.756 (0.895–3.444)
Post-natal adaptation	83 (4.6%)	27 (3.8%)	48 (5.2%)	8 (4.8%)	0.371	0.730 (0.450–1.185)	0.923 (0.427–1.996)
Bronchopulmonary dysplasia	48 (2.7%)	19 (2.7%)	23 (2.5%)	6 (3.6%)	0.735	1.024 (0.547–1.917)	1.397 (0.556–3.513)
Pneumonia	61 (3.4%)	18 (2.5%)	32 (3.5%)	11 (6.5%)	0.033^b^	0.724 (0.402–1.304)	2.056 (1.008–4.192)
Pneumothorax	39 (2.2%)	11 (1.5%)	25 (2.7%)	3 (1.8%)	0.244	0.555 (0.270–1.140)	0.695 (0.207–2.340)
Intraventricular haemorrhage	78 (4.3%)	29 (4.1%)	35 (3.8%)	14 (8.3%)	0.027^a^	1.047 (0.629–1.741)	2.215 (1.159–4.233)
Necrotising enterocolitis	62 (3.4%)	18 (2.5%)	37 (4.0%)	7 (4.2%)	0.212	0.652 (0.366–1.162)	0.988 (0.429–2.276)
Neonatal death	44 (2.4%)	17 (2.4%)	20 (2.2%)	7 (4.2%)	0.306	1.043 (0.538–2.019)	2.024 (0.832–4.923)
Sepsis	175 (9.7%)	58 (8.1%)	90 (9.8%)	27 (16.1%)	0.007^ab^	0.812 (0.574–1.150)	1.737 (1.086–2.777)
Hypothermia	52 (2.9%)	22 (3.1%)	23 (2.5%)	7 (4.2%)	0.465	1.284 (0.708–2.330)	1.648 (0.691–3.932)
Phototherapy	1054 (58.6%)	398 (55.6%)	532 (58.1%)	124 (73.8%)	< 0.001^ab^	0.925 (0.758–1.130)	1.997 (1.378–2.895)
Transient tachypnoea of the newborn	424 (23.6%)	156 (21.8%)	210 (22.9%)	58 (34.5%)	0.002^ab^	0.930 (0.734–1.178)	1.801 (1.261–2.571)
Ventilator support	238 (13.2%)	103 (14.4%)	103 (11.2%)	32 (19.0%)	0.011^a^	1.306 (0.973–1.754)	1.843 (1.187–2.861)
Continuous positive airway pressure	404 (22.4%)	138 (19.3%)	214 (23.4%)	52 (31.0%)	0.003^ab^	0.786 (0.617–1.002)	1.443 (1.003–2.075)
Positive pressure ventilation	235 (13.1%)	95 (13.3%)	107 (11.7%)	33 (19.6%)	0.019^a^	1.185 (0.880–1.596)	1.831 (1.185–2.829)
Intermediate care unit admission	1258 (69.9%)	518 (72.3%)	648 (70.7%)	92 (54.8%)	< 0.001^ab^	1.076 (0.864–1.339)	0.516 (0.368–0.725)
Neonatal intensive care unit admission	411 (22.8%)	150 (20.9%)	203 (22.2%)	58 (34.5%)	0.001^ab^	0.947 (0.745–1.204)	1.822 (1.275–2.604)
Respiratory distress syndrome	95 (5.3%)	42 (5.9%)	40 (4.4%)	13 (7.7%)	0.132	1.347 (0.861–2.106)	1.751 (0.910–3.366)
Persistent tachypnoea of the newborn	1 (0.1%)	1 (0.1%)	0 (0.0%)	0 (0.0%)	0.469	–	–
Length of hospital stay (days)	7 (1–179)	6 (1–179)	7 (1–179)	14 (1–179)	< 0.001^abc^	–	–
Mean neonatal weight (grams)	2208.5 ± 559.9	2305.5 ± 571.2	2181.7 ± 544.6	1942.4 ± 489.5	< 0.001^abc^	–	–
**Maternal**							
Gestational age at delivery (weeks of gestation)	34.4 ± 2.1	34.7 ± 2.0	34.4 ± 2.1	33.4 ± 2.0	< 0.001^abc^	–	–
Underlying disease							
Diabetes mellitus (DM)	29 (1.6%)	11 (1.5%)	15 (1.6%)	3 (1.8%)	0.970	0.937 (0.428–2.053)	1.092 (0.313–3.814)
Hypertension	107 (5.9%)	37 (5.2%)	50 (5.5%)	20 (11.9%)	0.003^ab^	0.944 (0.610–1.461)	2.341 (1.354–4.045)
Others	173 (9.6%)	60 (8.4%)	90 (9.8%)	23 (13.7%)	0.105	0.839 (0.596–1.182)	1.456 (0.891–2.378)
Length of hospital stay	6 (2–143)	4.5 (2–31)	7 (2–57)	14.5 (2–143)	< 0.001^abc^	–	–
Puerperal infection	13 (0.7%)	5 (0.7%)	6 (0.7%)	2 (1.2%)	0.749	1.067 (0.324–3.509)	1.827 (0.366–9.131)
Referral to other centres	36 (2.0%)	9 (1.3%)	20 (2.2%)	7 (4.2%)	0.045^b^	0.570 (0.258–1.260)	1.948 (0.810–4.682)

Chi-square test with multiple comparison by Bonferroni correction was used to compare the categorical outcome between groups

Kruskal-Wallis test with Dunn’s test correction for multiple comparison was used to compare the non-normally distributed continuous data between groups

One-way ANOVA with Bonferroni correction for multiple comparison was used to compare the normally distributed continuous data between groups

Multivariable logistic regression models were used to calculate the adjusted odds ratios and 95% confidence intervals (95% CI) to examine the association between steroid groups and adverse outcomes after adjusting for covariate (maternal age, BMI, morbidities)

^a^Statistically significant between 2 and 3

^b^Statistically significant between 1 and 3

^c^Statistically significant between 1 and 2

Compared with newborns whose mothers were administered more than 1 steroid course, newborns of mothers given less than 1 course were significantly less likely

- to have an APGAR score < 7 (at 1 minute)
- to have TTNB, pneumonia and sepsis
- to be admitted to the NICU
- to receive continuous positive airway pressure (CPAP)
- to receive phototherapy
- to need a prolonged hospital stay
- to be referred to other centres. (Table 3)

**Table 3:** Subgroup analysis by singleton pregnancy

Outcomes	Total (***N*** = 1585)	< 1 course (***n*** = 673): (1)	1 course (***n*** = 783): (2)	> 1 course (***n*** = 129): (3)	***P*** value	Adjusted odds ratio (95% CI) for < 1 course	Adjusted odds ratio (95% CI) for > 1 course
**Neonatal**							
APGAR score < 7 (1 min)	269 (17.0%)	96 (14.3%)	140 (17.9%)	33 (25.6%)	0.005^ab^	0.777 (0.584–1.034)	1.499 (0.962–2.336)
APGAR score < 7 (5 min)	78 (4.9%)	33 (4.9%)	35 (4.5%)	10 (7.8%)	0.279	1.139 (0.697–1.859)	1.899 (0.907–3.976)
Post-natal adaptation	66 (4.2%)	23 (3.4%)	39 (5.0%)	4 (3.1%)	0.271	0.703 (0.414–1.193)	0.627 (0.219–1.799)
Bronchopulmonary dysplasia	45 (2.8%)	18 (2.7%)	22 (2.8%)	5 (3.9%)	0.752	0.929 (0.487–1.769)	1.376 (0.504–3.761)
Pneumonia	54 (3.4%)	15 (2.2%)	29 (3.7%)	10 (7.8%)	0.005^ab^	0.609 (0.322–1.148)	2.516 (1.179–5.371)
Pneumothorax	30 (1.9%)	9 (1.3%)	19 (2.4%)	2 (1.6%)	0.301	0.552 (0.247–1.237)	0.757 (0.173–3.324)
Intraventricular haemorrhage	61 (3.8%)	23 (3.4%)	29 (3.7%)	9 (7.0%)	0.15	0.908 (0.515–1.600)	1.838 (0.840–4.023)
Necrotising enterocolitis	45 (2.8%)	12 (1.8%)	28 (3.6%)	5 (3.9%)	0.041^c^	0.515 (0.258–0.982)	1.002 (0.372–2.701)
Neonatal death	34 (2.1%)	14 (2.1%)	16 (2.0%)	4 (3.1%)	0.736	0.997 (0.479–2.076)	1.569 (0.504–4.884)
Sepsis	145 (9.1%)	48 (7.1%)	76 (9.7%)	21 (16.3%)	0.003^ab^	0.729 (0.499–1.065)	1.785 (1.050–3.034)
Hypothermia	41 (2.6%)	17 (2.5%)	18 (2.3%)	6 (4.7%)	0.294	1.144 (0.582–2.247)	2.033 (0.778–5.311)
Phototherapy	917 (57.9%)	370 (55.0%)	450 (57.5%)	97 (75.2%)	< 0.001^ab^	0.928 (0.752–1.145)	2.180 (1.420–3.349)
Transient tachypnoea of the newborn	344 (21.7%)	141 (21.0%)	163 (20.8%)	40 (31.0%)	0.028^ab^	1.001 (0.775–1.292)	1.719 (1.134–2.606)
Ventilator support	194 (12.2%)	89 (13.2%)	82 (10.5%)	23 (17.8%)	0.036^a^	1.281 (0.928–1.768)	1.861 (1.115–3.107)
Continuous positive airway pressure	320 (20.2%)	117 (17.4%)	166 (21.2%)	37 (28.7%)	0.008^b^	0.786 (0.603–1.025)	1.448 (0.948–2.210)
Positive pressure ventilation	201 (12.7%)	88 (13.1%)	86 (11.0%)	27 (20.9%)	0.007^a^	1.257 (0.912–1.731)	2.140 (1.313–3.486)
Intermediate care unit admission	1107 (69.8%)	491 (73.0%)	549 (70.1%)	67 (51.9%)	< 0.001^ab^	1.137 (0.903–1.432)	0.474 (0.323–0.695)
Neonatal intensive care unit admission	344 (21.7%)	133 (19.8%)	164 (20.9%)	47 (36.4%)	< 0.001^ab^	0.946 (0.730–1.225)	2.160 (1.444–3.232)
Respiratory distress syndrome	72 (4.5%)	34 (5.1%)	29 (3.7%)	9 (7.0%)	0.179	1.372 (0.824–2.283)	1.776 (0.812–3.886)
Persistent tachypnoea of the newborn	1 (0.1%)	1 (0.1%)	0 (0.0%)	0 (0.0%)	0.508	–	–
Length of hospital stay (days)	7 (1–179)	6 (1–179)	7 (1–179)	14 (1–179)	< 0.001^abc^	–	–
Mean neonatal weight (grams)	2245.5 ± 568.5	2327.9 ± 573.6	2219.1 ± 556.7	1975.6 ± 515.3	< 0.001^abc^	–	–
**Maternal**						–	–
Gestational age at delivery (weeks of gestation)	34.5 ± 2.1	34.8 ± 2.0	34.4 ± 2.1	33.4 ± 2.0	< 0.001^abc^	–	–
Underlying disease							
Diabetes mellitus (DM)	28 (1.8%)	11 (1.6%)	14 (1.8%)	3 (2.3%)	0.842	0.913 (0.412–2.204)	1.308 (0.371–4.616)
Hypertension	102 (6.4%)	37 (5.5%)	45 (5.7%)	20 (15.5%)	< 0.001^ab^	0.954 (0.610–1.493)	3.009 (1.712–5.288)
Others	153 (9.7%)	55 (8.2%)	78 (10.0%)	20 (15.5%)	0.034^b^	0.804 (0.560–1.155)	1.658 (0.975–2.821)
Length of hospital stay (days)	6 (2–143)	5 (2–31)	7 (2–56)	12 (2–143)	< 0.001^abc^	–	–
Puerperal infection	11 (0.7%)	5 (0.7%)	5 (0.6%)	1 (0.8%)	0.965	1.165 (0.336–4.040)	1.216 (0.141–10.490)
Referral to other centres	21 (1.3%)	5 (0.7%)	13 (1.7%)	3 (2.3%)	0.182	0.443 (0.157–1.250)	1.410 (0.396–5.019)

Chi-square test with multiple comparison by Bonferroni correction was used to compare the categorical outcome between groups

Kruskal-Wallis test with Dunn’s test correction for multiple comparison was used to compare the non-normally distributed continuous data between groups

One-way ANOVA with Bonferroni correction for multiple comparison was used to compare the normally distributed continuous data between groups

Multivariable logistic regression models were used to calculate the adjusted odds ratios and 95% confidence intervals (95% CI) to examine the association between steroid groups and adverse outcomes after adjusting for covariate (maternal age, BMI, morbidities)

^a^Statistically significant between 2 and 3

^b^Statistically significant between 1 and 3

^c^Statistically significant between 1 and 2

**Fig. 3:**
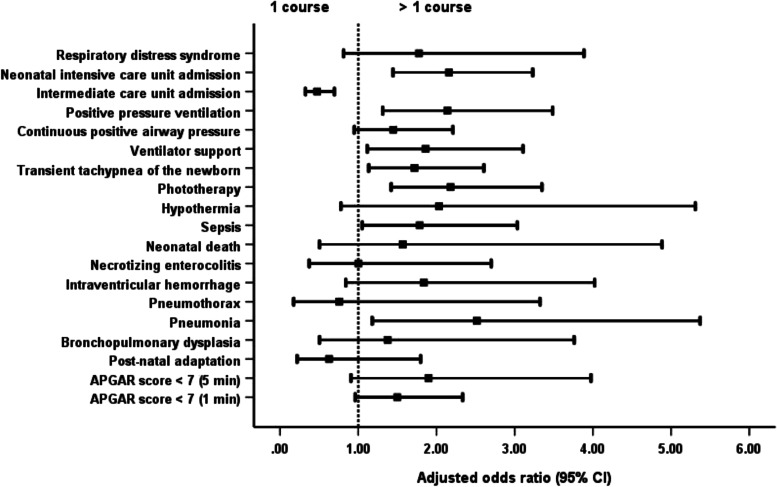
Comparison of singleton outcomes with the administration of 1 and >  1 course of dexamethasone. *For singleton, one course of dexamethasone has better neonatal and maternal outcomes than more than 1 course of dexamethasone administration

Compared with newborns whose mothers administered a single steroid course, singleton newborns of mothers given more than 1 course were more likely to have TTNB; to be admitted to the NICU; to need PPV, ventilatory support and phototherapy; and to need a prolonged hospital stay (Table 3; Fig. 3).

**Fig. 4:**
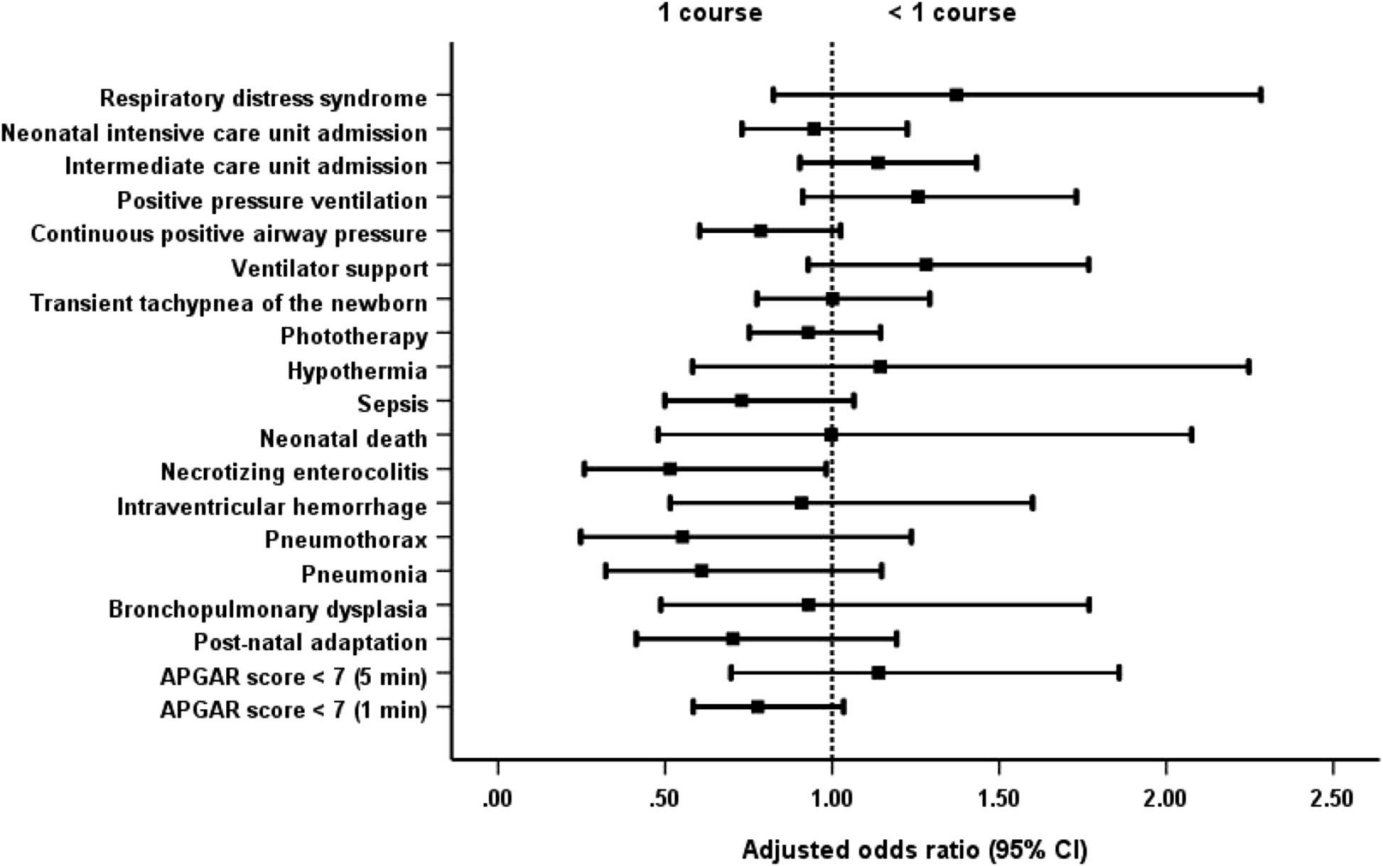
Comparison of singleton outcomes with the administration of 1 and < 1 course of dexamethasone. * For singleton, one course of dexamethasone has better outcome of necrotizing enterocoliti than partial course of steroid administration

In addition, singleton newborns of mothers given 1 course of steroids had NEC less frequently than those whose mothers were administered a partial course (Table 3; Fig. 4).

Compared with newborns whose mothers received more than 1 steroid course, singleton newborns of mothers given an incomplete course were less likely to have an APGAR score < 7 (at 1 minute); to have TTNB, pneumonia, and sepsis; to be admitted to the NICU; to receive CPAP and phototherapy; and to need a prolonged hospital stay (Table 3).

There were no statistically significant differences in the incidence of RDS or other adverse outcomes except for the length of hospital stay between 3- groups of mothers with twins neonates (Table 4).

**Table 4:** Subgroup analysis by twins pregnancy

Outcomes	Total (***N*** = 215)	< 1 course (***n*** = 43): (1)	1 course (***n*** = 133): (2)	> 1 course (***n*** = 39): (3)	***P*** value	Adjusted odds ratio (95% CI) for < 1 course	Adjusted odds ratio (95% CI) for > 1 course
**Neonatal**							
APGAR score < 7 (1 min)	32 (14.9%)	8 (18.6%)	17 (12.8%)	7 (17.9%)	0.543	1.543 (0.588–4.051)	1.552 (0.560–4.298)
APGAR score < 7 (5 min)	7 (3.3%)	1 (2.3%)	4 (3.0%)	2 (5.1%)	0.749	0.763 (0.078–7.449)	1.553 (0.250–9.638)
Post-natal adaptation	17 (7.9%)	4 (9.3%)	9 (6.8%)	4 (10.3%)	0.723	1.331 (0.382–4.639)	1480 (0.421–5.200)
Bronchopulmonary dysplasia	3 (1.4%)	1 (2.3%)	1 (0.8%)	1 (2.6%)	0.589	3.230 (0.181–57.63)	4.590 (0.250–84.38)
Pneumonia	7 (3.3%)	3 (7.0%)	3 (2.3%)	1 (2.6%)	0.305	2.821 (0.521–15.27)	1.343 (0.130–13.87)
Pneumothorax	9 (4.2%)	2 (4.7%)	6 (4.5%)	1 (2.6%)	0.855	0.858 (0.160–4.596)	0.452 (0.046–4.401)
Intraventricular haemorrhage	17 (7.9%)	6 (14.0%)	9 (6.8%)	2 (5.1%)	0.245	3.234 (0.976–10.72)	3.049 (0.865–10.75)
Necrotising enterocolitis	30 (14.0%)	10 (23.3%)	14 (10.5%)	6 (15.4%)	0.035^c^	2.484 (1.005–7.904)	0.714 (0.141–3.613)
Neonatal death	10 (4.7%)	3 (7.0%)	4 (3.0%)	3 (7.7%)	0.342	2.214 (0.450–10.90)	3.573 (0.704–18.13)
Sepsis	7 (3.3%)	3 (7.0%)	3 (2.3%)	1 (2.6%)	0.305	2.663 (1.051–6.746)	1.474 (0.507–4.279)
Hypothermia	11 (5.1%)	5 (11.6%)	5 (3.8%)	1 (2.6%)	0.091	2.911 (0.782–10.83)	0.659 (0.073–5.947)
Phototherapy	137 (63.7%)	28 (65.1%)	82 (61.7%)	27 (69.2%)	0.672	1.163 (0.562–2.407)	1.364 (0.629–2.959)
Transient tachypnoea of the newborn	80 (37.2%)	15 (34.9%)	47 (35.3%)	18 (46.2%)	0.442	0.967 (0.465–2.015)	1.567 (0.749–3.278)
Ventilator support	44 (20.5%)	14 (32.6%)	21 (15.8%)	9 (23.1%)	0.015^c^	2.878 (1.262–6.561)	1.746 (0.700–4.354)
Continuous positive airway pressure	84 (39.1%)	21 (48.8%)	48 (36.1%)	15 (38.5%)	0.329	1.801 (0.886–3.663)	1.156 (0.545–2.452)
Positive pressure ventilation	34 (15.8%)	7 (16.3%)	21 (15.8%)	6 (15.4%)	0.994	0.999 (0.386–2.587)	1.031 (0.377–2.802)
Intermediate care unit admission	151 (70.2%)	27 (62.8%)	99 (74.4%)	25 (64.1%)	0.227	0.570 (0.267–1.218)	0.594 (0.268–1.315)
Neonatal intensive care unit admission	67 (31.2%)	17 (39.5%)	39 (29.3%)	11 (28.2%)	0.412	1.699 (0.813–3.547)	0.956 (0.424–2.155)
Respiratory distress syndrome	23 (10.7%)	8 (18.6%)	11 (8.3%)	4 (10.3%)	0.162	2.688 (0.971–7.440)	1.410 (0.409–4.859)
Persistent tachypnoea of the newborn	–	–	–	–	–	–	–
Length of hospital stay (days)	13 (1–117)	13 (1–106)	13 (1–117)	14 (1–101)	0.865	–	–
Mean neonatal weight (grams)	1970.5 ± 443.4	1911.63 ± 387.3	2017.6 ± 461.2	1874.9 ± 426.2	0.131	–	–
**Maternal**							
Gestational age at delivery (weeks of gestation)	34.1 ± 2.1	33.8 ± 2.2	34.4 ± 2.1	33.5 ± 2.1	0.057	–	–
Underlying disease							
Diabetes mellitus (DM)	1 (0.5%)	0 (0.0%)	1 (0.8%)	0 (0.0%)	1.000	–	–
Hypertension	5 (2.3%)	0 (0.0%)	5 (3.8%)	0 (0.0%)	0.211	–	–
Others	20 (9.3%)	5 (11.6%)	12 (9.0%)	3 (7.7%)	0.843	1.327 (0.439–4.006)	0.840 (0.225–3.142)
Length of hospital stay	7 (2–124)	4 (3–22)	7 (2–57)	24 (3–124)	< 0.001^abc^	–	–
Puerperal infection	2 (0.9%)	0 (0.0%)	1 (0.8%)	1 (2.6%)	0.454	–	3.474 (0.212–56.85)
Referral to other centres	15 (7.0%)	4 (9.3%)	7 (5.3%)	4 (10.3%)	0.448	1.846 (0.513–6.639)	2.057 (0.570–7.431)

Chi-square test with multiple comparison by Bonferroni correction was used to compare the categorical outcome between groups

Kruskal-Wallis test with Dunn’s test correction for multiple comparison was used to compare the non-normally distributed continuous data between groups

One-way ANOVA with Bonferroni correction for multiple comparison was used to compare the normally distributed continuous data between groups

Multivariable logistic regression models were used to calculate the adjusted odds ratios and 95% confidence intervals (95% CI) to examine the association between steroid groups and adverse outcomes after adjusting for covariate (maternal age, BMI, morbidities)

^a^Statistically significant between 2 and 3

^b^Statistically significant between 1 and 3

^c^Statistically significant between 1 and 2

**Fig. 5:**
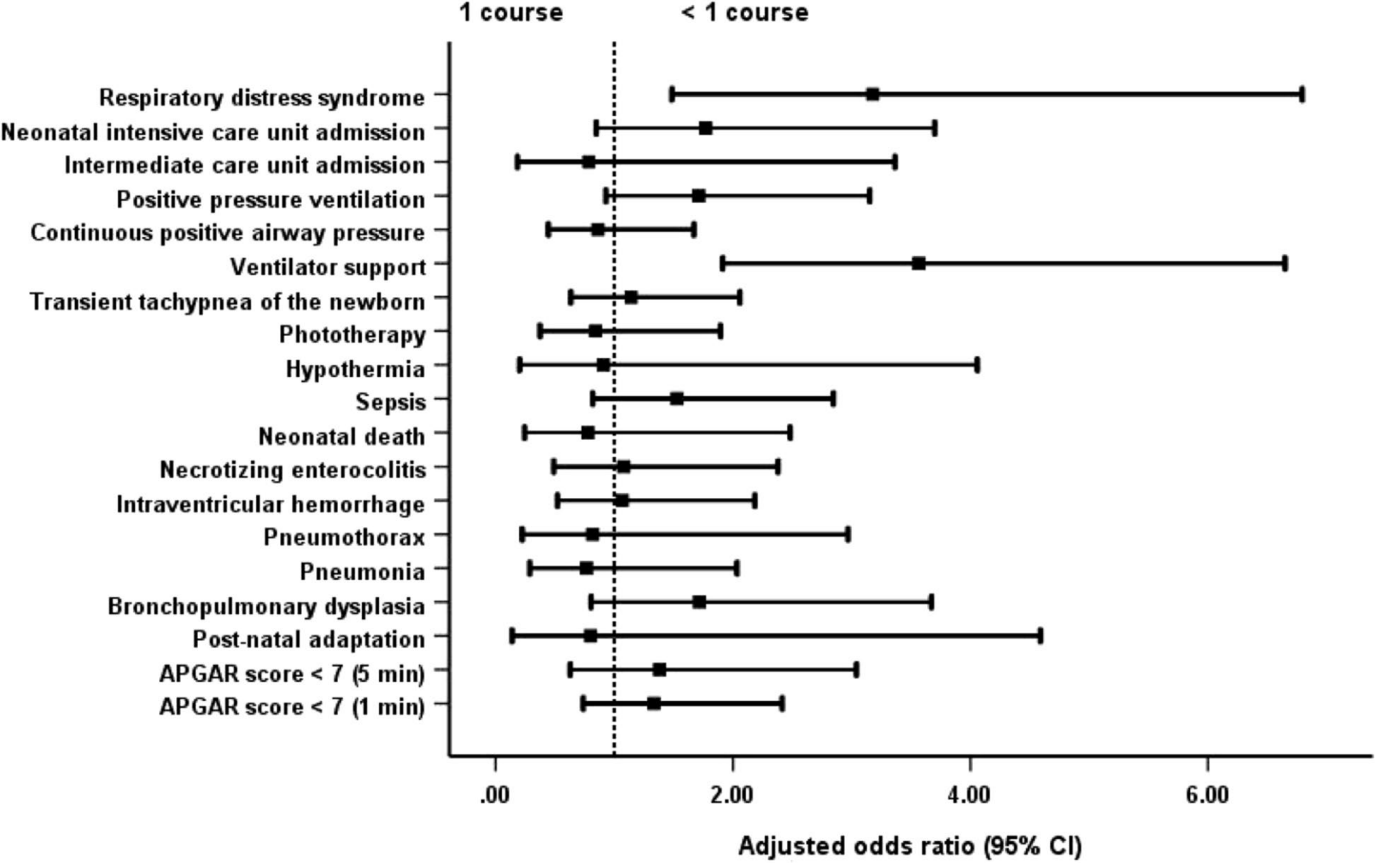
Comparison of very preterm outcomes with the administration of 1 and < 1 course of dexamethasone. * For very preterm, full course of dexamethasone has better outcomes of respiratory distress syndrome and ventilatory supports than partial course of dexamethasone administration

**Fig. 6:**
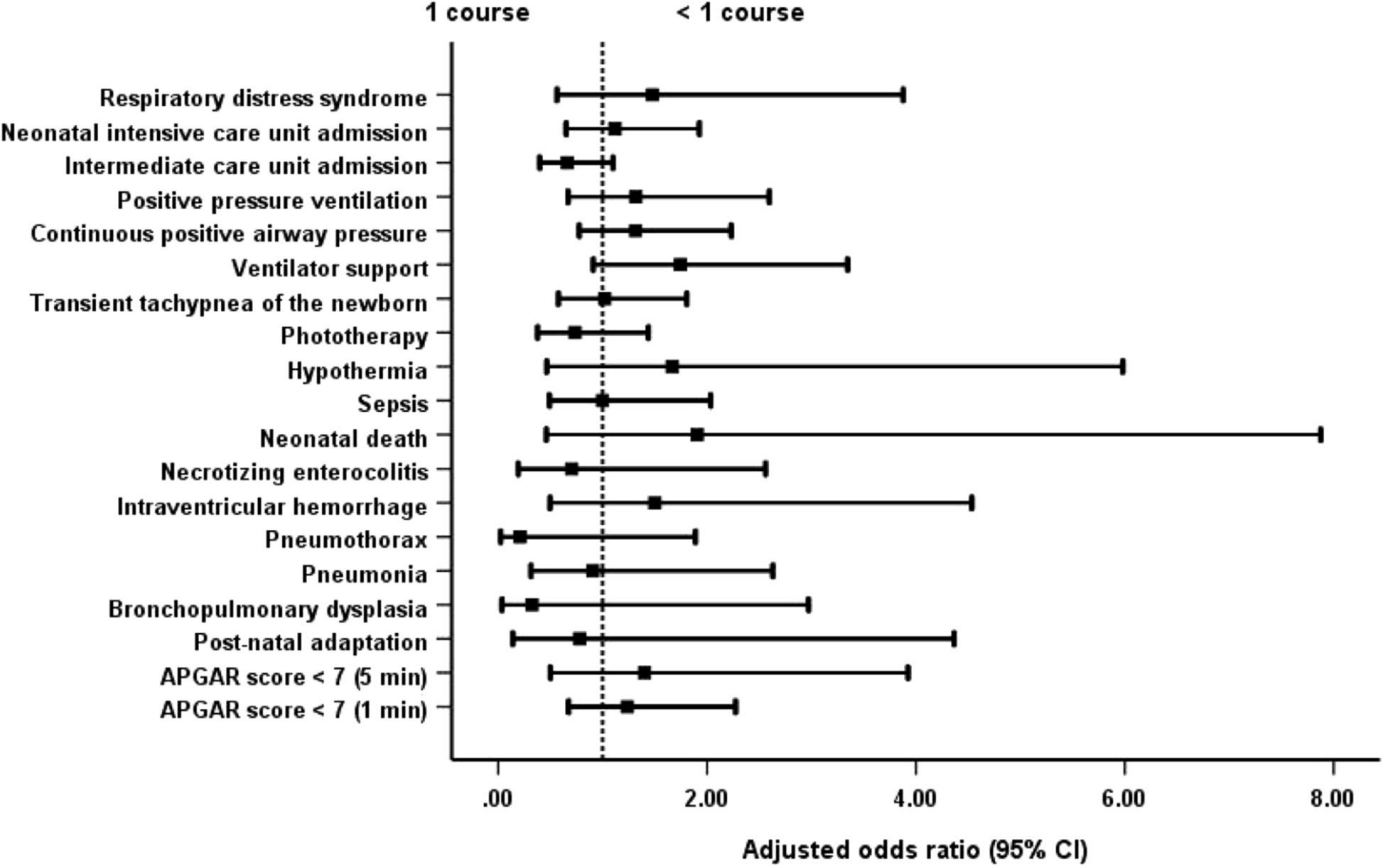
Comparison of moderate preterm outcomes with the administration of 1 and < 1 course of dexamethasone. * For moderate preterm, neonatal and maternal outcomes are not difference between the groups of full and partial course of dexamethasone administration

Very preterm newborns whose mothers received a partial course developed RDS and used ventilatory support more often than newborns whose mothers were given either a complete course or more than 1 course (Table 5, Fig. 5). Moderate preterm newborns whose mothers received a partial course had similar outcomes to the newborns whose mothers were given either a complete course or more than 1 course (Table 6, Fig. 6).

**Table 5:** Subgroup analysis by very preterm

Outcomes	Total (***N*** = 236)	< 1 course (***n*** = 77): (1)	1 course (***n*** = 123): (2)	> 1 course (***n*** = 36): (3)	***P*** value	Adjusted odds ratio (95% CI) for < 1 course	Adjusted odds ratio (95% CI) for > 1 course
**Neonatal**							
APGAR score < 7 (1 min)	105 (44.5%)	38 (49.4%)	50 (40.7%)	17 (47.2%)	0.438	1.334 (0.737–2.413)	1.192 (0.546–2.600)
APGAR score < 7 (5 min)	39 (16.5%)	14 (18.2%)	17 (13.8%)	8 (22.2%)	0.458	1.381 (0.628–3.039)	1.776 (0.674–4.682)
Post-natal adaptation	10 (4.2%)	2 (2.6%)	5 (4.1%)	3 (8.3%)	0.411	0.800 (0.139–4.590)	3.716 (0.757–18.25)
Bronchopulmonary dysplasia	40 (16.9%)	18 (23.4%)	17 (13.8%)	5 (13.9%)	0.202	1.717 (0.803–3.671)	0.966 (0.322–2.901)
Pneumonia	27 (11.4%)	7 (9.1%)	14 (11.4%)	6 (16.7%)	0.547	0.765 (0.288–2.032)	1.220 (0.403–3.697)
Pneumothorax	13 (5.5%)	4 (5.2%)	7 (5.7%)	2 (5.6%)	1.000	0.817 (0.225–2.969)	1.091 (0.205–5.794)
Intraventricular haemorrhage	53 (22.5%)	17 (22.1%)	26 (21.1%)	10 (27.8%)	0.718	1.066 (0.521–2.184)	1.394 (0.576–3.373)
Necrotising enterocolitis	39 (16.5%)	13 (16.9%)	22 (17.9%)	4 (11.1%)	0.660	1.079 (0.490–2.378)	0.572 (0.175–1.867)
Neonatal death	19 (8.1%)	5 (6.5%)	9 (7.3%)	5 (13.9%)	0.393	0.778 (0.244–2.480)	2.208 (0.648–7.526)
Sepsis	78 (33.1%)	30 (39.0%)	38 (30.9%)	10 (27.8%)	0.370	1.528 (0.820–2.844)	0.706 (0.295–1.693)
Hypothermia	9 (3.8%)	3 (3.9%)	5 (4.1%)	1 (2.8%)	1.000	0.908 (0.203–4.057)	0.824 (0.089–7.641)
Phototherapy	200 (84.7%)	63 (81.8%)	106 (86.2%)	31 (86.1%)	0.684	0.842 (0.374–1.895)	0.896 (0.289–2.777)
Transient tachypnoea of the newborn	117 (49.6%)	40 (51.9%)	59 (48.0%)	18 (50.0%)	0.862	1.141 (0.633–2.058)	1.047 (0.484–2.265)
Ventilator support	114 (48.3%)	53 (68.8%)	45 (36.6%)	16 (44.4%)	< 0.001^bc^	3.565 (1.912–6.650)	1.197 (0.543–2.641)
Continuous positive airway pressure	167 (70.8%)	54 (70.1%)	92 (74.8%)	21 (58.3%)	0.163	0.862 (0.444–1.671)	0.465 (0.205–1.054)
Positive pressure ventilation	86 (36.4%)	34 (44.2%)	38 (30.9%)	14 (38.9%)	0.162	1.711 (0.929–3.150)	1.362 (0.610–3.043)
Intermediate care unit admission	13 (5.5%)	3 (3.9%)	7 (5.7%)	3 (8.3%)	0.625	0.786 (0.184–3.364)	2.016 (0.442–9.184)
Neonatal intensive care unit admission	180 (76.3%)	63 (81.8%)	91 (74.0%)	26 (72.2%)	0.390	1.770 (0.847–3.699)	0.840 (0.349–2.017)
Respiratory distress syndrome	44 (18.6%)	23 (29.9%)	15 (12.2%)	6 (16.7%)	0.006^c^	3.177 (1.485–6.795)	1.115 (0.376–3.304)
Persistent tachypnoea of the newborn	–	–	–	–	–	–	–
Mean neonatal weight (grams)	1385.2 ± 371.6	1376.4 ± 362.5	1371.1 ± 384.1	1452.5 ± 349.0	0.498	–	–
**Maternal**							
Gestational age at delivery (weeks of gestation)	30.3 ± 1.1	30.3 ± 1.2	30.2 ± 1.2	30.5 ± 1.0	0.468	–	–
Underlying disease							
Diabetes mellitus (DM)	3 (1.3%)	1 (1.3%)	2 (1.6%)	0 (0.0%)	1.000	0.796 (0.071–8.930)	–
Hypertension	19 (8.1%)	8 (10.4%)	8 (6.5%)	3 (8.3%)	0.614	1.667 (0.598–4.642)	1.307 (0.328–5.206)
Others	22 (9.3%)	6 (7.8%)	13 (10.6%)	3 (8.3%)	0.791	0.715 (0.260–1.968)	0.769 (0.207–2.863)
Puerperal infection	2 (0.8%)	0 (0.0%)	2 (1.6%)	0 (0.0%)	0.658	–	–
Referral to other centres	18 (7.6%)	5 (6.5%)	9 (7.3%)	4 (11.1%)	0.704	0.880 (0.283–2.729)	1.583 (0.458–5.479)

Chi-square test with multiple comparison by Bonferroni correction was used to compare the categorical outcome between groups

Kruskal-Wallis test with Dunn’s test correction for multiple comparison was used to compare the non-normally distributed continuous data between groups

One-way ANOVA with Bonferroni correction for multiple comparison was used to compare the normally distributed continuous data between groups

Multivariable logistic regression models were used to calculate the adjusted odds ratios and 95% confidence intervals (95% CI) to examine the association between steroid groups and adverse outcomes after adjusting for covariate (maternal age, BMI, morbidities)

^a^Statistically significant between 2 and 3

^b^Statistically significant between 1 and 3

^c^Statistically significant between 1 and 2

**Table 6:** Subgroup analysis by moderate preterm

Outcomes	Total (***N*** = 317)	< 1 course (***n*** = 103): (1)	1 course (***n*** = 161): (2)	> 1 course (***n*** = 53): (3)	***P*** value	Adjusted odds ratio (95% CI) for < 1 course	Adjusted odds ratio (95% CI) for > 1 course
**Neonatal**							
APGAR score < 7 (1 min)	78 (24.6%)	26 (25.2%)	37 (23.0%)	15 (28.3%)	0.712	1.236 (0.672–2.274)	1.311 (0.619–2.773)
APGAR score < 7 (5 min)	20 (6.3%)	8 (7.8%)	10 (6.2%)	2 (3.8%)	0.672	1.400 (0.500–3.924)	0.594 (0.105–3.352)
Post-natal adaptation	9 (2.8%)	2 (1.9%)	5 (3.1%)	2 (3.8%)	0.817	0.781 (0.140–4.365)	1.719 (0.294–10.05)
Bronchopulmonary dysplasia	7 (2.2%)	1 (1.0%)	5 (3.1%)	1 (1.9%)	0.684	0.323 (0.035–2.971)	0.846 (0.088–8.133)
Pneumonia	19 (6.0%)	6 (5.8%)	10 (6.2%)	3 (5.7%)	1.000	0.905 (0.312–2.628)	1.047 (0.268–4.089)
Pneumothorax	9 (2.8%)	1 (1.0%)	7 (4.3%)	1 (1.9%)	0.278	0.209 (0.023–1.887)	0.363 (0.028–4.678)
Intraventricular haemorrhage	18 (5.7%)	7 (6.8%)	7 (4.3%)	4 (7.5%)	0.575	1.499 (0.496–4.534)	1.679 (0.436–6.466)
Necrotising enterocolitis	14 (4.4%)	4 (3.9%)	8 (5.0%)	2 (3.8%)	0.934	0.702 (0.192–2.560)	1.055 (0.202–5.524)
Neonatal death	10 (3.2%)	5 (4.9%)	4 (2.5%)	1 (1.9%)	0.572	1.905 (0.461–7.876)	0.417 (0.019–9.147)
Sepsis	51 (16.1%)	15 (14.6%)	26 (16.1%)	10 (18.9%)	0.784	0.998 (0.490–2.034)	1.371 (0.594–3.165)
Hypothermia	15 (4.7%)	5 (4.9%)	6 (3.7%)	4 (7.5%)	0.525	1.667 (0.465–5.981)	3.166 (0.767–13.07)
Phototherapy	267 (84.2%)	82 (79.6%)	136 (84.5%)	49 (92.5%)	0.112	0.735 (0.376–1.436)	1.933 (0.623–5.995)
Transient tachypnoea of the newborn	94 (29.7%)	28 (27.2%)	43 (26.7%)	23 (43.4%)	0.056	1.019 (0.575–1.805)	2.199 (1.114–4.341)
Ventilator support	60 (18.9%)	24 (23.3%)	25 (15.5%)	11 (20.8%)	0.275	1.744 (0.909–3.346)	1.763 (0.769–4.041)
Continuous positive airway pressure	118 (37.2%)	41 (39.8%)	55 (34.2%)	22 (41.5%)	0.507	1.314 (0.774–2.231)	1.424 (0.735–2.757)
Positive pressure ventilation	58 (18.3%)	20 (19.4%)	26 (16.1%)	12 (22.6%)	0.515	1.317 (0.669–2.595)	1.772 (0.784–4.004)
Intermediate care unit admission	163 (51.4%)	46 (44.7%)	91 (56.5%)	26 (49.1%)	0.162	0.661 (0.397–1.099)	0.739 (0.388–1.405)
Neonatal intensive care unit admission	109 (34.4%)	34 (33.0%)	50 (31.1%)	25 (47.2%)	0.095	1.118 (0.649–1.925)	2.238 (1.155–4.336)
Respiratory distress syndrome	23 (7.3%)	9 (8.7%)	10 (6.2%)	4 (7.5%)	0.735	1.477 (0.563–3.878)	1.168 (0.340–4.010)
Persistent tachypnoea of the newborn	–	–	–	–	–	–	–
Mean neonatal weight (grams)	1929.8 ± 377.0	1928.5 ± 391.6	1938.1 ± 333.6	1906.8 ± 468.7	0.871	–	–
**Maternal**							
Gestational age at delivery (weeks of gestation)	33.1 ± 0.6	33.0 ± 0.6	33.1 ± 0.6	33.0 ± 0.6	0.170	–	–
Underlying disease							
Diabetes mellitus (DM)	4 (1.3%)	1 (1.0%)	2 (1.2%)	1 (1.9%)	1.000	0.779 (0.070–8.707)	1.529 (0.136–17.21)
Hypertension	23 (7.3%)	6 (5.8%)	8 (5.0%)	9 (17.0%)	0.012	1.183 (0.398–3.514)	3.912 (1.425–10.74)
Others	24 (7.6%)	8 (7.8%)	11 (6.8%)	5 (9.4%)	0.812	1.148 (0.446–2.958)	1.420 (0.470–4.293)
Puerperal infection	5 (1.6%)	1 (1.0%)	2 (1.2%)	2 (3.8%)	0.471	0.779 (0.070–8.707)	3.118 (0.428–22.70)
Referral to other centres	9 (2.8%)	2 (1.9%)	5 (3.1%)	2 (3.8%)	0.817	0.618 (0.118–3.245)	1.224 (0.230–6.500)

Chi-square test with multiple comparison by Bonferroni correction was used to compare the categorical outcome between groups

Kruskal-Wallis test with Dunn’s test correction for multiple comparison was used to compare the non-normally distributed continuous data between groups

One-way ANOVA with Bonferroni correction for multiple comparison was used to compare the normally distributed continuous data between groups

Multivariable logistic regression models were used to calculate the adjusted odds ratios and 95% confidence intervals (95% CI) to examine the association between steroid groups and adverse outcomes after adjusting for covariate (maternal age, BMI, morbidities)

^a^Statistically significant between 2 and 3

^b^Statistically significant between 1 and 3

^c^Statistically significant between 1 and 2

For the late preterm group, neonatal and maternal outcomes were not difference between 3 groups of dexamethasone administration. (Table 7).

**Table 7:** Subgroup analysis by late preterm

Outcomes	Total (***N*** = 1247)	< 1 course (***n*** = 536): (1)	1 course (***n*** = 632): (2)	> 1 course (***n*** = 79): (3)	***P*** value	Adjusted odds ratio (95% CI) for < 1 course	Adjusted odds ratio (95% CI) for > 1 course
**Neonatal**							
APGAR score < 7 (1 min)	118 (9.5%)	40 (7.5%)	70 (11.1%)	8 (10.1%)	0.107	0.664 (0.441–0.979)	0.849 (0.389–1.854)
APGAR score < 7 (5 min)	26 (2.1%)	12 (2.2%)	12 (1.9%)	2 (2.5%)	0.884	1.224 (0.543–2.756)	1.270 (0.276–5.855)
Post-natal adaptation	64 (5.1%)	23 (4.3%)	38 (6.0%)	3 (3.8%)	0.354	0.730 (0.428–1.244)	0.573 (0.171–1.916)
Bronchopulmonary dysplasia	1 (0.1%)	0 (0.0%)	1 (0.2%)	0 (0.0%)	0.615	–	–
Pneumonia	15 (1.2%)	5 (0.9%)	8 (1.3%)	2 (2.5%)	0.467	0.731 (0.236–2.264)	2.227 (0.453–10.94)
Pneumothorax	17 (1.4%)	6 (1.1%)	11 (1.7%)	0 (0.0%)	0.368	0.639 (0.233–1.748)	–
Intraventricular haemorrhage	7 (0.6%)	5 (0.9%)	2 (0.3%)	0 (0.0%)	0.294	2.800 (0.538–14.58)	–
Necrotising enterocolitis	9 (0.7%)	1 (0.2%)	7 (1.1%)	1 (1.3%)	0.151	0.165 (0.020–1.354)	0.965 (0.110–8.506)
Neonatal death	15 (1.2%)	7 (1.3%)	7 (1.1%)	1 (1.3%)	0.952	1.183 (0.405–3.457)	1.156 (0.135–9.938)
Sepsis	46 (3.7%)	13 (2.4%)	26 (4.1%)	7 (8.9%)	0.013^b^	0.573 (0.290–1.130)	2.232 (0.921–5.406)
Hypothermia	28 (2.2%)	14 (2.6%)	12 (1.9%)	2 (2.5%)	0.703	1.503 (0.684–3.304)	1.221 (0.264–5.634)
Phototherapy	587 (47.1%)	253 (47.2%)	290 (45.9%)	44 (55.7%)	0.257	1.073 (0.850–1.355)	1.459 (0.907–2.347)
Transient tachypnoea of the newborn	213 (17.1%)	88 (16.4%)	108 (17.1%)	17 (21.5%)	0.531	0.934 (0.684–1.275)	1.389 (0.775–2.488)
Ventilator support	64 (5.1%)	26 (4.9%)	33 (5.2%)	5 (6.3%)	0.848	0.923 (0.543–1.569)	1.248 (0.469–3.324)
Continuous positive airway pressure	119 (9.5%)	43 (8.0%)	67 (10.6%)	9 (11.4%)	0.277	0.712 (0.475–1.067)	1.107 (0.524–2.337)
Positive pressure ventilation	91 (7.3%)	41 (7.6%)	43 (6.8%)	7 (8.9%)	0.737	1.177 (0.752–1.843)	1.321 (0.568–3.071)
Intermediate care unit admission	1082 (86.8%)	469 (87.5%)	550 (87.0%)	63 (79.7%)	0.159	1.018 (0.719–1.442)	0.609 (0.333–1.113)
Neonatal intensive care unit admission	122 (9.8%)	53 (9.9%)	62 (9.8%)	7 (8.9%)	0.959	1.027 (0.697–1.515)	0.876 (0.384–1.998)
Respiratory distress syndrome	28 (2.2%)	10 (1.9%)	15 (2.4%)	3 (3.8%)	0.531	0.762 (0.338–1.717)	1.729 (0.483–6.192)
Persistent tachypnoea of the newborn	1 (0.1%)	1 (0.2%)	0 (0.0%)	0 (0.0%)	0.515	–	–
Length of hospital stay (days)	5 (1–179)	5 (1–179)	5 (1–166)	7 (2–179)	< 0.001^ab^	–	–
Mean neonatal weight (grams)	2441.2 ± 437.9	2508.0 ± 444.5	2413.3 ± 426.9	2210.4 ± 377.9	< 0.001^abc^	–	–
**Maternal**							
Gestational age at delivery (weeks of gestation)	35.6 ± 0.9	35.7 ± 0.9	35.5 ± 0.9	35.0 ± 0.9	< 0.001^abc^	–	–
Underlying disease							
Diabetes mellitus (DM)	22 (1.8%)	9 (1.7%)	11 (1.7%)	2 (2.5%)	0.879	0.964 (0.396–2.344)	1.466 (0.319–6.739)
Hypertension	65 (5.2%)	23 (4.3%)	34 (5.4%)	8 (10.1%)	0.084	0.789 (0.459–1.356)	1.982 (0.883–4.448)
Others	127 (10.2%)	46 (8.6%)	66 (10.4%)	15 (19.0%)	0.017^b^	0.805 (0.542–1.196)	2.010 (1.084–3.726)
Length of hospital stay (days)	6 (2–124)	5 (2–18)	6 (2–57)	13 (2–124)	< 0.001^abc^		
Puerperal infection	6 (0.5%)	4 (0.7%)	2 (0.3%)	0 (0.0%)	0.466	2.368 (0.432–12.98)	–
Referral to other centres	9 (0.7%)	2 (0.4%)	6 (0.9%)	1 (1.3%)	0.429	0.391 (0.079–1.944)	1.338 (0.159–11.26)

Chi-square test with multiple comparison by Bonferroni correction was used to compare the categorical outcome between groups

Kruskal-Wallis test with Dunn’s test correction for multiple comparison was used to compare the non-normally distributed continuous data between groups

One-way ANOVA with Bonferroni correction for multiple comparison was used to compare the normally distributed continuous data between groups

Multivariable logistic regression models were used to calculate the adjusted odds ratios and 95% confidence intervals (95% CI) to examine the association between steroid groups and adverse outcomes after adjusting for covariate (maternal age, BMI, morbidities)

^a^Statistically significant between 2 and 3

^b^Statistically significant between 1 and 3

^c^Statistically significant between 1 and 2

## Discussion

Compared with newborns whose mothers were given a single course of dexamethasone, preterm neonates of women administered more than 1 dexamethasone course

- suffered more frequently from RDS, TTNB, sepsis and IVH;
- were admitted more often to the NICU; and
- were more likely to need PPV, CPAP, ventilator support and phototherapy.

Another course of dexamethasone was given after 7 days if indications of the need for steroids were still present or as a rescue course. The guidelines of the American College of Obstetricians and Gynecologists for preterm labour management were followed [37].

Our results support many investigations that compared the benefits and risks of single versus several steroid courses before delivery. Many studies concluded that multiple steroid courses do not reduce the incidence of RDS, IVH, NEC and neonatal sepsis or the need for PPV [28, 42, 43]. Multiple courses increase the incidence of short- and long-term effects. They are infection, neonatal mortality, maternal and foetal adrenal suppression, maternal infection, impaired glucose tolerance, osteoporosis and possibly neonatal chronic lung disease. In addition, neonatal birth weight and head circumference are reduced [30, 44, 45].

Our study also presented the differences in gestational age at delivery and neonatal weight between the three groups. The group with multiple steroid courses had the lowest neonatal weight and gestational age at delivery which may correlated with more adverse neonatal outcomes. While the group who received a partial course of dexamethasone had the highest neonatal weight and gestational age at delivery and outcomes were not different from the group with a full course dexamethasone who had lower neonatal weight and gestational age at delivery. Those results were a limitation of our study in the data interpretation. Because neonate with a lower gestational age at delivery and lower neonatal weight tend to have more adverse neonatal outcomes.

However, other research found that multiple steroid courses did not have significant adverse effects in the form of reduced neonatal birth weight, decreased head circumference, or neonatal and maternal infections [46, 47]. The US National Institutes of Health and Goldenberg and Wright concluded that, on balance, current evidence is not sufficient to support the benefits of administering multiple courses of steroids [48, 49].

We did not find any significant differences in the incidence of RDS and other adverse outcomes in the newborns of women who received a partial course of dexamethasone and neonates of women who received a full course. The guidelines of the Royal College of Obstetricians and Gynaecologists of 2022 state that giving steroids—even 1 dose or 24 hours before delivery—helps reduce breathing difficulties in preterm neonates [50]. Therefore, steroid administration should not be delayed for women at risk of preterm delivery. However, a full course of steroid therapy has been shown to significantly reduce the incidence of RDS in preterm neonates [51].

There were generally no differences in the adverse outcomes of preterm neonates whose mothers received a single dexamethasone course and newborns whose mothers had an incomplete course. The exception was NEC, which occurred less frequently among mothers given a complete course. Animal studies have shown that a steroid course can reduce NEC incidence [52, 53].

Late preterm birth accounts for 75% of all preterm births and is associated with higher morbidity and mortality rates than for full-term infants. Although steroids can reduce mild respiratory difficulties, such as TTNB, these conditions often improve spontaneously. In addition, steroids may be associated with neonatal hypoglycaemia and short- and long-term effects. There are not sufficient studies on the long-term benefits or harm of steroids. Steroid administration is not recommended in pregnant women with a gestational age greater than 34 weeks. The administration should be considered on an individual basis and should only be carried out for women prone to preterm delivery and likely to benefit from steroids [54–57].

In addition, pregnant women with late preterm may not be prescribed tocolytic drugs for inhibition of labour. This may increase the chances of not receiving the full course of dexamethasone and may also affect breathing difficulties in preterm newborns.

The twin pregnancies (215/1585; 13.5%) in our 3 study groups had no difference in RDS. Most twin pregnancies have a very high chance of very-to-moderate preterm delivery. Only one-third of preterm deliveries in twin pregnancies are late preterm. There is currently no evidence to support the efficacy of steroids in reducing RDS in twins delivered during late preterm, except for evidence of increased risk of foetal growth restriction [54, 58, 59]. Further studies on steroid administration in late-preterm twin pregnancies are needed.

Steroids were found to have a greater benefit in reducing RDS in very-to-moderate preterm infants than in late preterm infants [50]. Our research found that with very-to-moderate preterm infants, a full course of dexamethasone reduced the need for RDS ventilator support better than a partial course of dexamethasone. Chen-Yu et al. [60] also concluded that an incomplete corticosteroid course was associated with an increased incidence of RDS compared with a full course of therapy. Therefore, inhibition of uterine contraction for at least 48 hours for the full course of steroids is beneficial in very-to-moderate preterm labour. Ay et al. reported that 1 and 2 steroid courses resulted in no statistically significant differences in the incidence of RDS and the need for ventilator therapy [61]. Although multiple courses of corticosteroid therapy do not increase or decrease the risk of death or disability of infants at 5 years of age compared with a single course, there is still a lack of conclusive evidence of long-term benefits. Multiple courses are not recommended for women at risk of preterm delivery [62]. If there is no emergency condition for delivery, inhibition of labour at least 48 hours for full course of dexamethasone in very to moderate preterm results in the most beneficial to preterm newborns.

The current obstetrician guidelines aim to deliver birth within 7 days of antenatal corticosteroid administration [63]. A single course of steroids positively affected feeding and growth outcomes in very-low-birth-weight preterm infants born at 28 to 32 weeks of gestation. However, a similar phenomenon was not observed in a repeat course of the steroid regimen [64]. Many courses of steroid administration are not recommended in cases of preterm premature rupture of membranes. The outcomes of the newborns are not improved, and multiple courses have been associated with an increased risk of chorioamnionitis [43]. Prenatal corticosteroid therapy significantly reduces the incidence and severity of RDS in preterm neonates. It should be administered to women between 24 and 34 weeks of gestation who are in preterm labour [65].

Multiple courses of steroids in lambs improved lung function, decreased birth weight [66], reduced brain weight and delayed myelination of the optic nerve [23]. Earlier observational studies on monkeys, rats and rabbits showed evidence of adrenal suppression, growth retardation and poor neurodevelopment, which raises concerns about repeated antenatal corticosteroid use [67]. Repeat courses of steroid administration in twin pregnancies were associated with a reduction in birth weight [68]. Moreover, repeat courses of steroid administration in preterm delivery at 32 weeks of gestation improved lung function but with a higher rate of cerebral palsy in children; however, there were no statistically significant differences [32, 69]. Murphy et al. reported that multiple courses of corticosteroids were ineffective in improving preterm outcomes and were associated with a decrease in weight, length and head circumference at birth [70]. Multiple courses of steroid administration should be concerned and reconsidered for the short and long term adverse outcomes of babies.

The Australasian Collaborative Trial of Repeat Doses of Steroids (ACTORDS) enrolled 982 women at less than 32 weeks of gestation. The findings, published in 2006, demonstrated short-term neonatal benefits of repeat courses of antenatal corticosteroids compared with women receiving placebo (RDS, 33% vs 41%; relative risk, 0.82; 95% CI, 0.71–0.95; *P* = 0.01) [71].

The ACTORDS trial showed that neurosensory disability or body size of children at 2 years of age were not different between the two groups, which repeated a single corticosteroid injection every 7 days and every 14 days [33]. The trial of Peltoniemi et al. was discontinued because of increased RDS and the need for surfactant therapy in the rescue treatment group before delivery [72]. A meta-analysis reported that repeat doses of prenatal corticosteroids reduced the occurrence and severity of neonatal lung disease and the risk of severe health problems in the first few weeks of life and were associated with a decrease in birth weight and head circumference. However, there is not sufficient evidence about the long-term benefits and risks [73].

A single course of antenatal corticosteroids for women with preterm labour is very beneficial. Still, the decision to proceed with steroid administration is problematic in cases of high-risk pregnant women who do not deliver after 7 days and still have a risk of preterm delivery. Such women are those with twin pregnancies, a previous history of preterm birth, a history of cervical surgeries, and foetal growth restriction. Forty per cent of twins are born at less than 37 weeks of gestation [74].

Antenatal corticosteroid therapy should be restricted to a single course of treatment administered at the optimal time, as judged by an experienced clinician for the given clinical circumstances. The current evidence indicates that the benefits of steroids are best achieved if a complete course is given at least 24 hours before delivery. In practice, however, the determination of the exact time of delivery is difficult; therefore, antenatal corticosteroid administration requires careful consideration. Unfortunately, many randomised controlled trials will continue to confuse, leading to variations in practice. Repeat courses of corticosteroids cannot currently be recommended in the absence of conclusive studies on their long-term adverse outcomes.

## Conclusions

Compared with neonates of women given 1 dexamethasone course, preterm singleton newborns whose mothers were administered multiple courses had an increased RDS incidence (without statistical significance) and several adverse outcomes (with significance). The negative outcomes were the development of TTNB, IVH, and sepsis; admission to the NICU and the intermediate care unit; and the need for ventilator support and phototherapy. Very-to-moderate preterm newborns whose mothers received a full dexamethasone course had a statistically significant decrease in RDS compared with neonates of women given an incomplete course.

The limitation of our study is the gestational age at delivery and neonatal weight was lowest in the group of multiple courses and that this could explain some of their higher risk for adverse outcomes.

## Data Availability

The data that support the findings of this study are available from Faculty of Medicine, Siriraj Hospital, Mahidol University but restrictions apply to the availability of these data, which were used under license for the current study, and so are not publicly available. Data are however available from the authors upon reasonable request and with permission of Faculty of Medicine, Siriraj Hospital, Mahidol University.
